# Neurovascular protection of alisol A on cerebral ischemia mice through activating the AKT/GSK3β pathway

**DOI:** 10.18632/aging.205151

**Published:** 2023-10-26

**Authors:** Huihong Li, Caiyun Zhang, Yangjie Zhou, Yunfei Deng, Xiaoqing Zheng, Xiehua Xue

**Affiliations:** 1 The Affiliated Rehabilitation Hospital, Fujian University of Traditional Chinese Medicine, Fuzhou, China; 2 The Zhangpu Hospital of Traditional Chinese Medicine, Zhangzhou, Fujian, China; 3 College of Rehabilitation Medicine, Fujian University of Traditional Chinese Medicine, Fuzhou, China; 4 Key Laboratory of Cognitive Rehabilitation of Fujian Province, Fuzhou, China

**Keywords:** alisol A, cerebral ischemia, hippocampus, neurovascular, AKT/GSK3β

## Abstract

Alisol A, a triterpene isolated from Alisma Orientale, has been shown to exhibit anti-inflammatory effects and vascular protection. This study was designed to observe the effect of alisol A on cerebral ischemia (CI)-induced neurovascular dysfunction in the hippocampus and to further explore the potential mechanisms. The results showed that alisol A treatment improved the neurological deficits and cognitive impairment of CI mice. Alisol A reduced gliosis and improved neuronal/glial metabolism. Accordingly, alisol A inhibited inflammatory factors IL-6 and IL-1β induced by overactivation of astrocytes and microglia, thus protecting the neurovasculature. Furthermore, alisol A promoted the survival of neurons by decreasing the ratio of Bax/Bcl-2, and protected brain microvascular endothelial cells (BMECs) by upregulating the expression of ZO-1, Occludin and CD31. The phosphorylation of protein kinase B (AKT) and glycogen synthase kinase 3β (GSK3β) increased after treatment with alisol A. To explore the underlying mechanism, AKT was inhibited. As expected, the neurovascular protection of alisol A above was eliminated by AKT inhibition. The present study primarily suggested that alisol A could exert neurovascular protection in the hippocampus of CI mice by activating the AKT/GSK3β pathway and may potentially be used for the treatment of CI.

## INTRODUCTION

Stroke is the third most common contributor to disability [1]. The incidence of CI accounts for approximately 87% of all stroke cases [2]. Stroke often leads to severe motor dysfunction and cognitive impairment. Cognitive impairment after stroke is highly prevalent (15%–70%) [3]. The prevalence of stroke increases during aging; therefore, CI occurs more often among elderly individuals. In recent years, stroke-related cognitive impairment has seriously endangered the health of the elderly [4]. Effective treatment of neurovascular dysfunction of CI has attracted increasing attention. The neurovascular function of the hippocampus plays an important role in the pathogenesis of CI [5]. CI induces inflammation, apoptosis, glial overactivation, blood brain barrier (BBB) and neuron disruption in the hippocampus, contributing to cognitive decline [6]. The neurovascular integrity in the hippocampus is critical for learning and memory and susceptible to ischemia and hypoxia [5]. Therefore, neurovascular protection in the hippocampus is extremely important for the treatment of CI.

AKT, a serine-threonine kinase, plays a vital role in cell death/survival. Phosphorylated/activated AKT is decreased in focal ischemia with aging [7]. Activated AKT exhibits a wide range of phosphorylation cascade events and phosphorylates GSK3β. GSK3β plays a pivotal role in regulating the balance between proinflammatory and anti-inflammatory effects. GSK3β is inactivated by PI3K/AKT activation, which protects the brain by promoting angiogenesis, neurogenesis, anti-apoptosis, and anti-inflammation. Activation of the AKT/GSK3β signaling pathway enhanced neurovascular restoration against ischemic brain injury [8]. Furthermore, inhibition of AKT/GSK3β was involved in ischemia-induced cognitive impairment [9]. These findings suggest that the AKT/GSK3β pathway plays a key role in neurovascular protection and cognitive improvement.

Traditional Chinese medicine (TCM) has multiple targets for improving CI [10]. In recent years, the pharmacology of Alisma Orientale has attracted increasing attention. Alisma Orientale are used to treat cardiovascular diseases, inflammatory diseases, and prevent atherosclerosis [11]. Triterpenoids isolated from the dried rhizomes of Alisma species have been shown to have antiproliferative, antiallergic, antibacterial and antiviral properties [12]. Alisol A is one of the main single component extracts of Alisma Orientale and shows significant anti-inflammatory and anti-atherosclerosis effects. It has been reported that alisol A is a multitargeted agent that exerts vascular protection by inhibiting inflammation [13]. The protein-protein interaction network analysis showed that AKT is one of the core therapeutic targets of Alisma Orientale [14]. The PI3K/AKT pathway is the central mechanism of Alisma Orientale [12]. These findings suggest that AKT may be one of the key targets of alisol A in the treatment of CI-induced neurovascular destruction. Therefore, we speculated that alisol A might play a role in neurovascular protection and alleviate cognitive decline by activating the AKT/GSK3β pathway.

## MATERIALS AND METHODS

### Animals

C57BL/6J mice (*n* = 50) (male, weighing 22–25 g, 12 weeks) were obtained from GemPharmatech (Jiangsu, China). All procedures, protocols, treatments and sampling were approved by the Ethics Committee of Fujian University of Traditional Chinese Medicine and were performed in strict accordance with the animal care and use guidelines of the National Institutes of Health. Permit number: SCXK (Su) 2018-0008). Animal ethics approval number: 2020091. The CI model was produced as described previously [15]. Mice were anesthetized with 1% pentobarbital sodium (0.3 ml/100 g), and both carotid arteries were occluded for 20 minutes. Then, the occlusion was removed to restore cerebral blood flow. Mice in the sham group received the same surgical procedure, but the carotid arteries were not occluded.

### Experimental design

Alisol A (Shanghai Yuanye Bio-Technology, Shanghai, China, B21638) was suspended in a solvent made up of lotus root powder and normal saline and then fully stirred in a warm bath to disperse evenly, and administered by gavage once a day for 7 consecutive days after CI (30 mg/kg). The AKT inhibitor GSK690693 (Apexbio, TX, USA, Catalog No. A5072) was injected intraperitoneally into mice at a dosage of 30 mg/kg [16] for 3 days and dissolved in DMSO. After CI, mice were randomly divided into four groups: sham-operated group (sham group, *n* = 10), cerebral ischemia group (CI group, *n* = 10), cerebral ischemia + alisol A group (CI+AA group, *n* = 10), and cerebral ischemia + alisol A+AKT inhibitor group (CI+AA+inh group, *n* = 10). The sham group and CI group were administered equal amounts of lotus root powder.

### Modified neurological severity score (mNSS)

mNSS was used to assess animals’ motor, sensory, balance, and reflex behaviors in each group (*n* = 10), which was performed by an investigator who was blinded to the grouping at 1, 3, 5, and 7 days after CI. mNSS scores ranged from 0 to 18 points. 0, no deficit; 1–6, mild deficit; 7–12, moderate deficit; 13–18, severe deficit.

### Morris water maze (MWM)

MWM was performed after 7 days of intragastric intervention, when the body capacity and wound of the mice had recovered completely. The first stage was MWM training (1–4th day). Mice in each group (*n* = 10) were dropped into the water from four different quadrants to swim to the target platform that was set before. Escape latency and total distance (the time and distance required for mice to find the target platform) were recorded in the MWM training stage. Mice were required to stay on the platform for 15 s once they reached the target platform for more than 60 s. The second stage is the MWM test (the 5th day). The platform was removed, and swimming trajectories to reach the platform were captured. The number of target quadrant crossings and time spent in the quadrant were recorded in the MWM test stage.

### New object recognition test (NORT)

The ability of mice to recognize new objects was evaluated by NORT. Each mouse was allowed to explore the open-field arena (40 × 40 × 40 cm) for 5 minutes without objects during the adaptation stage. 24 hours after habituation, two identical objects were placed on opposite corners, and mice were allowed to explore the two objects for 5 minutes. After 24 hours, one of the identical objects was replaced with a novel object, and mice were placed in the arena again to explore novel objects and familiar objects for 5 minutes. The discrimination ratio was calculated as the ratio of the number of explorations of the novel object to the number of explorations of the two identical objects. The movements of mice in the arena were recorded with a digital camera. The objects and the open-field arena were cleaned with 70% ethanol between each trial.

### Magnetic resonance spectroscopy (MRS)

MRS provides information on neuronal/glial metabolism. The hippocampal CA1 area was selected as the region of interest, with a 1 mm × 1 mm × 1 mm size. MRS was acquired using a PRESS sequence, and the parameters of MRS were TR = 1500 ms, TE = 144 ms, number of averages = 256, and voxel = 5 mm × 4 mm × 4 mm. The reference values for each metabolite are as follows: N-acetyl aspartate (NAA): 2.02 ppm; creatine (Cr): 3.05 ppm; choline (Cho): 3.2 ppm; myo-inositol (MI): 3.56 ppm. Cr was used as an internal reference to calculate the relative levels of other metabolites. TOPSPIN (V3.1, Bruker Biospin, Ettlingen, Germany) of the MRI instrument was used to analyze related images and data.

### Transmission electron microscopy (TEM)

TEM was used to detect changes in the neurovascular ultrastructure after CI. The hippocampal CA1 region was cut into 1 mm × 1 mm × 1 mm blocks and postfixed in 4% paraformaldehyde and 2.5% glutaraldehyde at 4°C. Samples were washed with phosphate buffer saline 3 times and then immersed in 1% osmium tetroxide in 0.1 M phosphate buffer for 2 hours at 4°C. Samples were cut into ultrathin sections and stained with 3% lead citrate. Then, TEM (Hitachi, HT7800, Tokyo, Japan) was used to observe and capture the neurovascular ultrastructure.

### Nissl staining

After being fixed with 4% paraformaldehyde, tissues were embedded in paraffin and then cut into serial 4 μm-thick coronal sections. After dewaxing, the sections were stained with Nissl staining solution (Solarbio, cat# G1435, Beijing, China) for 30 min at 37°C. Subsequently, the sections were rinsed in graded ethanol and cleared with xylene. After drying thoroughly, the sections were cover slipped with neutral resin. Images were acquired with a Nikon microscope (Nikon, Model Eclipse Ci-L, 718345, Tokyo, Japan). The neuronal morphology of the hippocampal CA1 region was observed at 400X magnification. The number of surviving neurons was counted by ImageJ software (version 6.0; Motic China Group Co., Ltd., Xiamen, China) and calculated by averaging the number in the same fields of three sections. Three mice in each group were included in the statistical analysis.

### Immunofluorescence staining

Immunofluorescence staining was applied to evaluate the expression of GFAP, Iba1, NeuN, and CD31 in the hippocampal CA1 region. Brain tissues were sliced into 4 μm-thick coronal sections and reacted with antibodies against GFAP (1:500, Proteintech, IL, USA, Cat No.: 16825-1-AP), Iba1 (1:500, Proteintech, Cat No.: 10904-1-AP), NeuN (1:500, Proteintech, Cat No.: 26975-1-AP), and CD31 (1:500, Cell Signaling Technology, MA, USA) overnight at 4°C. The corresponding secondary antibodies of Alexa Fluor 594 (1:500, Cat# ab150080) were employed and reacted for 1 h. Then, DAPI was added (Beyotime, Shanghai, China, Cat No. P0131) before being photographed. Finally, images were captured using Leica Microsystems under a 200X microscope. The fluorescence intensity was analyzed according to previous literature [17] and measured in the ipsilateral hippocampal CA1 region by ImageJ software.

### Western blot

The primary antibodies we used were as follows: ZO-1 (1:1000, Proteintech, Cat No. 21773-1-AP), Occludin (1:1000, Proteintech, Cat No. 66378-1-Ig), IL-6 (1:800, Proteintech, Cat. No. 66146-1-Ig), IL-1β (1:1000, Proteintech, Cat No. 16806-1-AP), BAX (1:1000, Proteintech, Cat No. 50599-2-Ig), Bcl-2 (1:1000, Proteintech, Cat No. 26593-1-AP), AKT (1:1000, Cell Signaling Technology, Cat#4691), P-AKT (Ser473, 1:1000, Cell Signaling Technology, Cat#4060), GSK3β (1:1000, Cell Signaling Technology, Cat# 12456S), P-GSK3β (1:1000, Cell Signaling Technology, Cat# 9323S), and GAPDH (1:8000, Proteintech, Cat# 60004-1-lg). The appropriate secondary antibodies (1:8000, Cat No. SA00001-1, Anti-Mouse; Cat No. SA00001-2 Anti-Rabbit) were incubated at room temperature for 1 hour. Finally, data were detected and analyzed by an Image Lab system (Bio-Rad, CA, USA, 721BR18682).

### Statistical analysis

All statistical analyses were performed using SPSS 23.0. Data confirmed to have a normal distribution were analyzed by one-way ANOVA followed by the LSD or Games-Howell post hoc test and are presented as the mean ± SD. Values of *P* < 0.05 were considered statistically significant. Independent experiments were repeated at least 3 times.

## RESULTS

### Alisol A improved neurological deficits after CI

**Figure 1:**
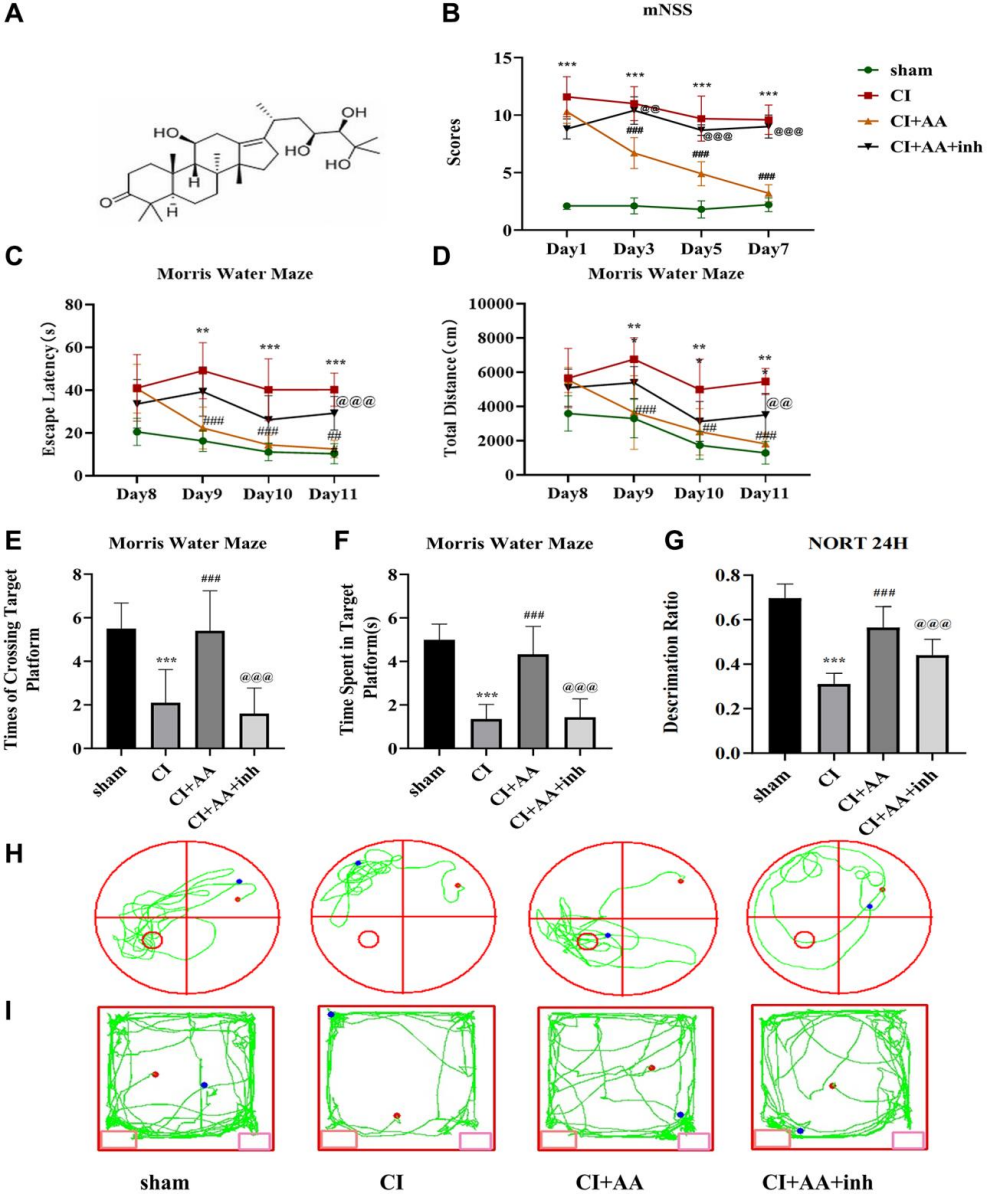
**Alisol A improved neurological deficits and cognitive impairment after CI.** (**A**) The molecular formula of alisol A. (**B**) Neurobehavioral function of mice was evaluated by mNSS at 1, 3, 5, and 7 days after CI, *n* = 10. (**C**) Escape latency of each group in the MWM training stage. (**D**) Total distance traveled by each group in the MWM training stage. (**E**) Frequency of crossing the target quadrant of each group in the MWM test. (**F**) Time spent in the target quadrant of each group in the MWM test. (**G**) Discrimination ratio of each group in NORT. (**H**) Representative tracking images in the MWM test. (**I**) Representative tracking images in NORT, *n* = 10. Data are shown as the mean ± SD. ^**^P < 0.01, ^***^P < 0.001 compared with the sham group, ^##^*P* < 0.01, ^###^*P* < 0.001 compared with the CI group, ^@@^*P* < 0.01, ^@@@^*P* < 0.001 compared with the CI+AA group. Note: sham-operated group (sham group); cerebral ischemia group (CI group); cerebral ischemia + alisol A (CI+AA group); cerebral ischemia + alisol A+AKT inhibitor group (CI+AA+inh group).

The molecular formula of alisol A is shown in Figure 1A. CI was established, and the detection of cerebral blood flow was described in a previous study [18]. The protective effects of alisol A against CI were examined by evaluating motor and sensory dysfunction according to mNSS (Figure 1B). Compared with the sham group, the CI group exhibited obvious neurological dysfunction. A significant improvement in mNSS was observed in mice treated with alisol A, while the result was reversed by the AKT inhibitor. The data above revealed that alisol A alleviated neurological deficits.

### Alisol A improved cognitive impairment after CI

The learning and memory abilities of mice were evaluated by the MWM. The escape latency and total distance traveled showed a decreasing trend in the MWM training stage (Figure 1C, 1D). On Days 2–4 of the training stage, the escape latency of mice in the CI group was significantly prolonged (*P* < 0.01, *P* < 0.001, *P* < 0.001), and the total distance traveled was increased (*P* < 0.01, *P* < 0.01, *P* < 0.01) compared with the sham group. Compared with the CI group, alisol A treatment shortened the escape latency (*P* < 0.001, *P* < 0.01, *P* < 0.01) and decreased the total distance traveled (*P* < 0.001, *P* < 0.01, *P* < 0.001). Although the difference between the CI+AA group and the CI+AA+inh group was not significant on Days 1–3, it still indicated that the AKT inhibitor weakened the effect of alisol A on the spatial memory and learning abilities of mice. The differences in escape latency and total distance traveled were significant on Day 4 (*P* < 0.001, *P* < 0.01). On Day 5 of the test stage, compared with the sham group, there were significant reductions in the number of target platform crossings (*P* < 0.001) and time spent in the target quadrant (*P* < 0.001) in the CI group. The results were improved by the treatment of alisol A (*P* < 0.001, *P* < 0.001). However, the effect of alisol A was reversed by the AKT inhibitor (*P* < 0.001, *P* < 0.001) (Figure 1E, 1F). The trajectory diagram of mice in each group was significantly different in the number of target platform crossings (Figure 1H).

The recognition memory of mice was detected by NORT (Figure 1G). Compared with the sham group, the discrimination ratio in the CI group was significantly decreased (*P* < 0.001), increased in the CI+AA group (*P* < 0.001), and decreased in the CI+AA+inh group (*P* < 0.001). The trajectory diagram of the mice is shown in Figure 1I. The data above revealed that alisol A effectively alleviated cognitive impairment.

### Alisol A regulated hippocampal metabolism

**Figure 2:**
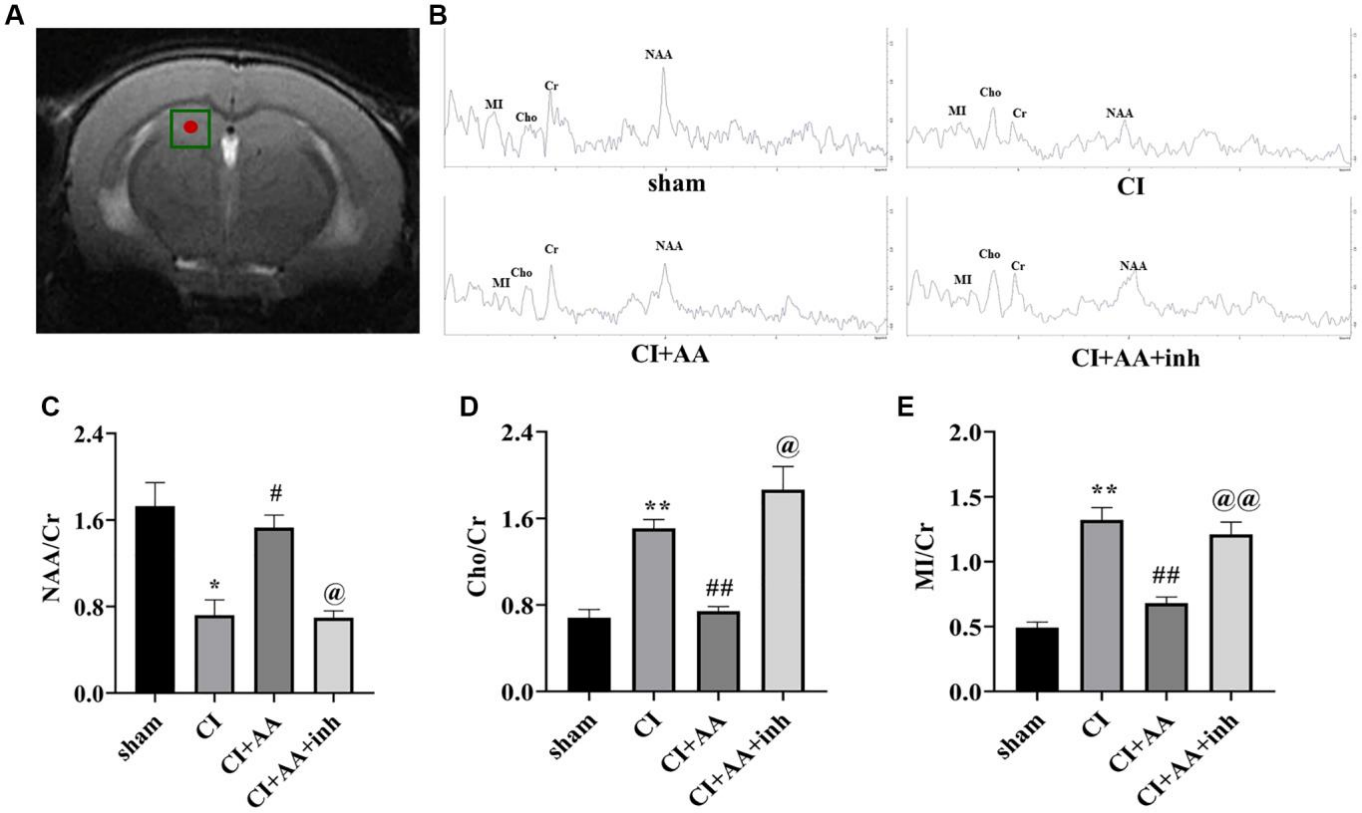
**Alisol A regulated hippocampal metabolism.** (**A**) The hippocampal CA1 region of mice was chosen as the region of interest. (**B**) Representative spectral image of the hippocampal CA1 region of mice. (**C**) Quantitative analysis of NAA/Cr. (**D**) Quantitative analysis of Cho/Cr. (**E**) Quantitative analysis of MI/Cr, *n* = 6. Data are presented as the mean ± SD, ^*^P < 0.05, ^**^P < 0.01 compared with the sham group, ^#^*P* < 0.05, ^##^*P* < 0.01 compared with the CI group, ^@^*P* < 0.05, ^@@^*P* < 0.01 compared with the CI+AA group.

The relative levels of Cr, NAA, Cho and MI in the CA1 region of the hippocampus were detected by MRS (Figure 2A). Figure 2B shows a representative spectral image of the hippocampal CA1 region of mice. NAA is a specific biochemical marker to assess neuronal viability/integrity, and Cho and MI levels indicate information about glial metabolism [19]. The NAA/Cr ratio of the CI group was significantly reduced (*P* < 0.05), and the NAA/Cr ratio was increased by alisol A treatment (*P* < 0.05) and decreased by the AKT inhibitor (*P* < 0.05) (Figure 2C). The results indicated that alisol A treatment improved the destruction of neurons, while the effect was reversed by an AKT inhibitor. Compared with the sham group, Cho/Cr and MI/Cr ratios in the CI group increased with statistically significant (*P* < 0.01, *P* < 0.01). Alisol A decreased the ratios of Cho/Cr and MI/Cr (*P* < 0.01, *P* < 0.01), and the inhibitor reversed the results (*P* < 0.05, *P* < 0.01) (Figure 2D, 2E). MI and Cho have been considered putative glial markers, suggesting that alisol A reduced gliosis and improved neuronal/glial metabolism after CI.

### Alisol A protected the neuronal ultrastructure in the hippocampal CA1 region

**Figure 3:**
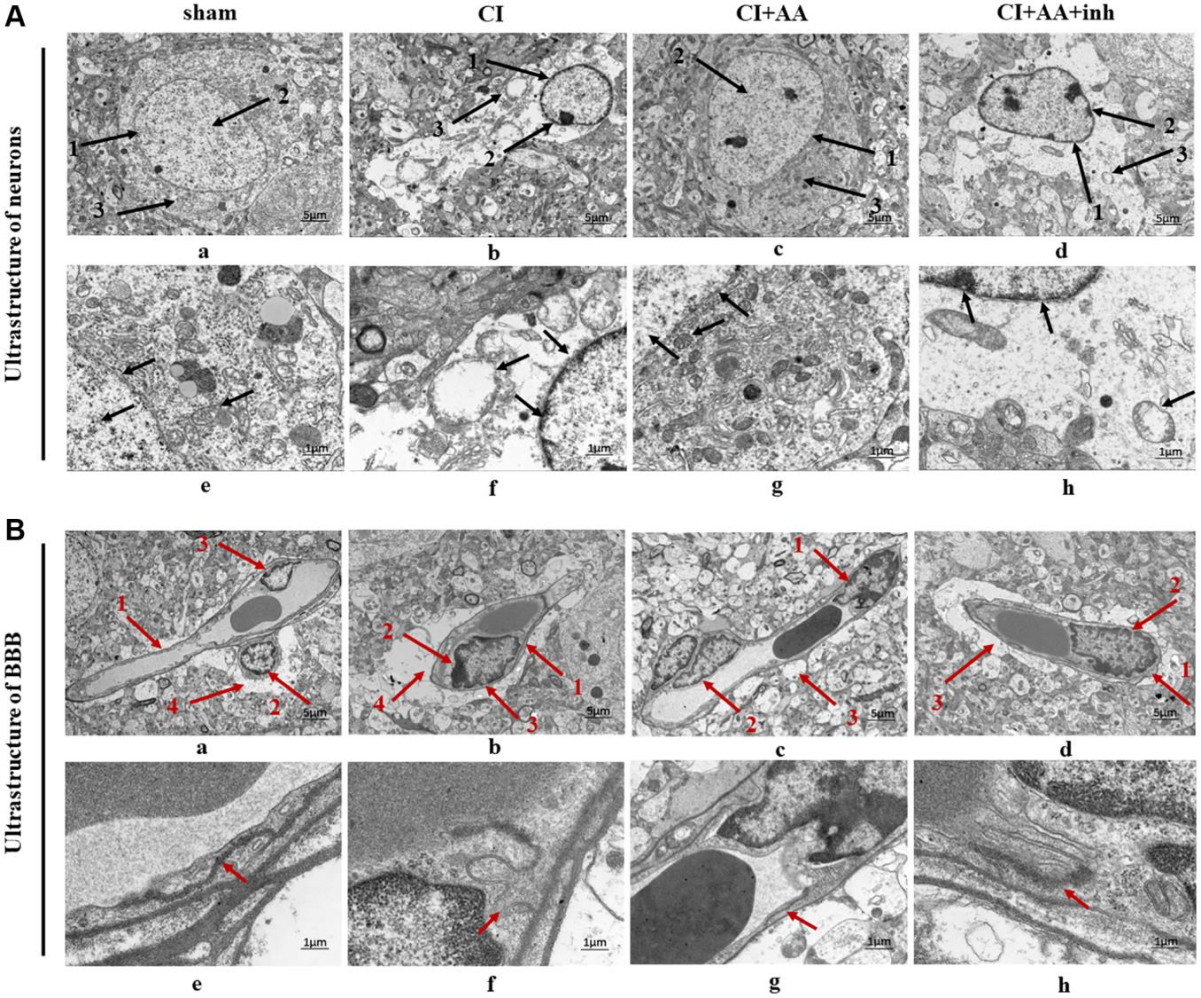
**Alisol A protected the neurovascular ultrastructure in the hippocampal CA1 region.** (**A**) Changes in neuronal ultrastructure are indicated by black arrows. (**a**) 1: The nuclear membrane structure of the neurons was intact and clear; 2: Chromatin distributed evenly; 3: Normal structural organelles were abundant within the cytoplasm. (**b**) 1: Incoherent nuclear membrane structure; 2: Chromatin condensation, decreased and edge aggregation; 3: Most of the organelles disappeared, and the cytoplasm was empty. (**c**) 1: The nuclear membrane is relatively complete, and the edge is clear; 2: More normal chromatin; 3: The organelles can be seen in the cytoplasm. (**d**) 1: Discontinuous and indistinct nuclear membrane structure; 2: Less chromatin; 3: Partial dissolution and disappearance of intracytoplasmic organelles. (**a**–**d**) Magnification of the microphotograph is 2500X. Scale bar is 5 μm; (**e**–**h**) Magnifications of the microphotograph are 8000X. Scale bar is 1 μm. (**B**) Changes in BBB ultrastructure as indicated by the red arrow. (**a**) 1: Clear capillary structures; 2: Close contact of the endothelial cells with the basement membrane; 3: Continuous endothelial cells; 4: End-feet of astrocyte surrounding the basal membrane. (**b**) 1: Capillary structure enhancement; 2: Edema fluid between the endothelial cells and the basement membrane; 3: Discontinuity of endothelium. 4: Marked edema of astrocytic end-foot processes and vacuolization. (**c**) 1: The edema between endothelial cells and the basal layer was weakened; 2: The endothelium was more continuous; 3: Vacuolization of astrocytic end-foot process edema was alleviated. (**d**) 1: There is edema between the endothelial cells and the basement membrane; 2: The endothelium is discontinuous and interrupted; 3: Perivascular astrocytic end-foot process edema was obvious and still showed vacuolar changes. (**a**–**d**) Magnification of the microphotograph is 2500X. Scale bar is 5 μm; (**e**–**h**) Magnifications of the microphotograph are 8000X. Scale bar is 1 μm. *n* = 3.

Neurons had an intact nuclear membrane structure and regular nuclear morphology in the sham group (Figure 3A (a, e)). Chromatin was distributed evenly within the nucleus. The nuclei with irregular shapes were slightly dissolved, and the chromatin showed condensation and edge aggregation and an incoherent nuclear membrane structure in the CI group (Figure 3A (b, f)). Moreover, some organelles were destroyed and even disappeared. The nuclear membrane was relatively intact, and the edge was clear after treatment with alisol A (Figure 3A (c, g)). In the inhibitor group (Figure 3A (d, h)), neurons with discontinuous and indistinct nuclear membrane structures had irregular morphology. Partial dissolution and disappearance of intracytoplasmic organelles can still be seen.

### Alisol A protected the BBB ultrastructure in the hippocampal CA1 region

The sham group showed clear capillary brain structure, and the basal layer was in close contact with the endothelial cells and had a clear level of clarity (Figure 3B (a, e)). The CI group was characterized by discontinuity of the endothelium and TJs and swelling of the astrocytes (Figure 3B (b, f)). The capillary wall was thickened, and there was edema between the endothelial cells and basement membrane. Alisol A treatment improved the swelling of astrocyte end-feet, and the basal layer was in close contact with the endothelial cells, while there were still some vacuolar changes around the blood vessels (Figure 3B (c, g)). There was edema between the microvascular endothelial layer and basal lamina and discontinuity of the endothelium after inhibitor intervention (Figure 3B (d, h)).

### Alisol A inhibited the expression of astrocytes, microglia, IL-6 and IL-1β after CI

**Figure 4:**
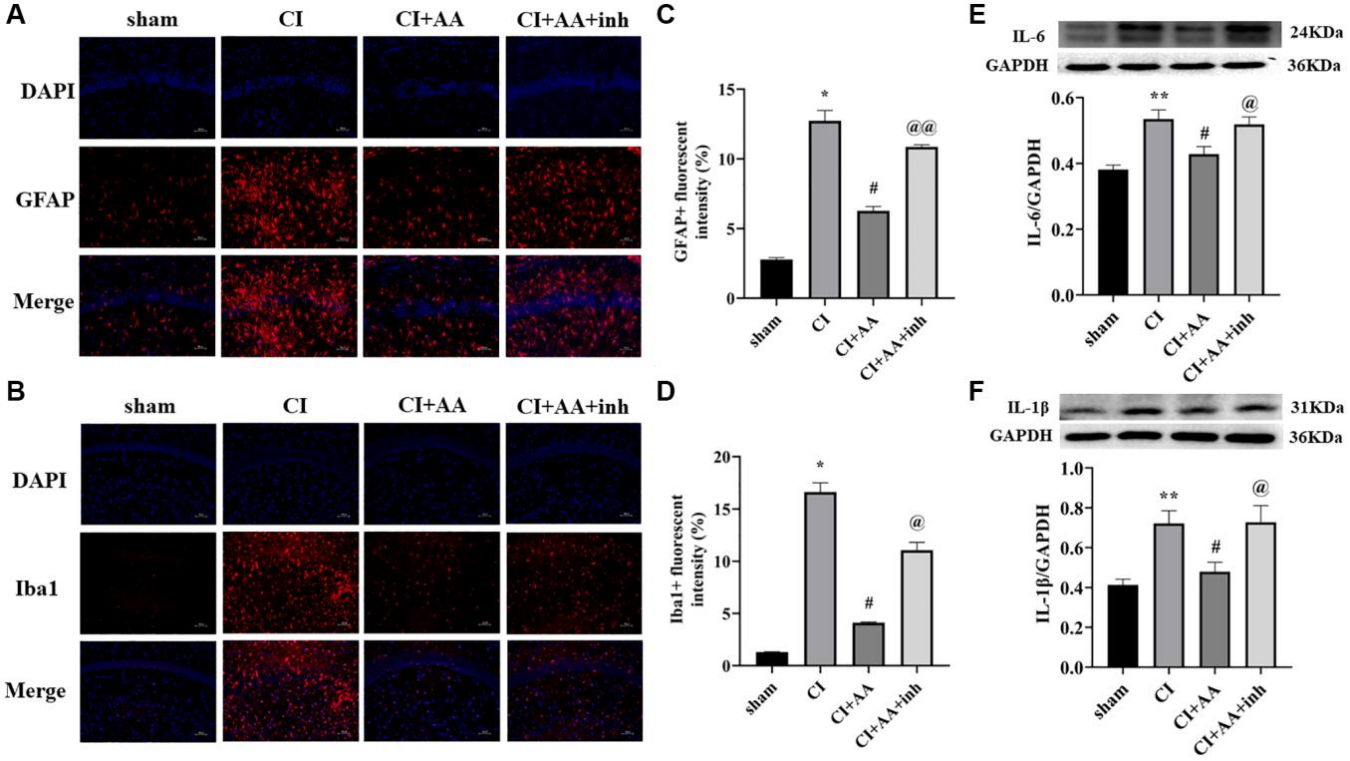
**Alisol A inhibited the expression of astrocytes, microglia, IL-6 and IL-1β after CI.** (**A**) Representative image of GFAP in the hippocampal CA1 region by immunofluorescence staining. (**B**) Representative image of Iba1 in the hippocampal CA1 region by using immunofluorescence staining. 200×, scale bar is 100 μm. (**C**) Quantitative analysis of GFAP fluorescence intensity. (**D**) Quantitative analysis of Iba1 fluorescence intensity, *n* = 3. (**E**) Western blot showing the expression of IL-6 and quantitative analysis of the ratio of IL-6 to GAPDH. (**F**) Western blot showing the expression of IL-1β and quantitative analysis of the ratio of IL-1β to GAPDH, *n* = 4. Data are shown as the mean ± SD. ^*^P < 0.05, ^**^P < 0.01 compared with the sham group, ^#^*P* < 0.05 compared with the CI group, ^@^*P* < 0.05, ^@@^*P* < 0.01 compared with the CI+AA group.

The number of glial cells increased after CI, inducing inflammation in the brain. Astrocytes and microglia (Figure 4A, 4C) were overactivated in the CI group compared with the sham group (*P* < 0.05, *P* < 0.05). Alisol A inhibited the overexpression of GFAP and Iba1 (*P* < 0.05, *P* < 0.05), and the AKT inhibitor restored the overactivation of GFAP and Iba1 (*P* < 0.01, *P* < 0.05) (Figure 4B, 4D). Overactivated glial cells produce proinflammatory factors that further damage brain injury. The expression of IL-6 and IL-1β was increased in the CI group (*P* < 0.01, *P* < 0.01). Alisol A markedly reduced IL-6 and IL-1β levels (*P* < 0.05, *P* < 0.05). Compared with alisol A treatment, the AKT inhibitor increased the expression of IL-6 and IL-1β (*P* < 0.05, *P* < 0.05) (Figure 4E, 4F). The results revealed that alisol A inhibited glial activation and reduced the production of inflammatory factors.

### Alisol A upregulated the expression of CD31, ZO-1 and Occludin after CI

**Figure 5:**
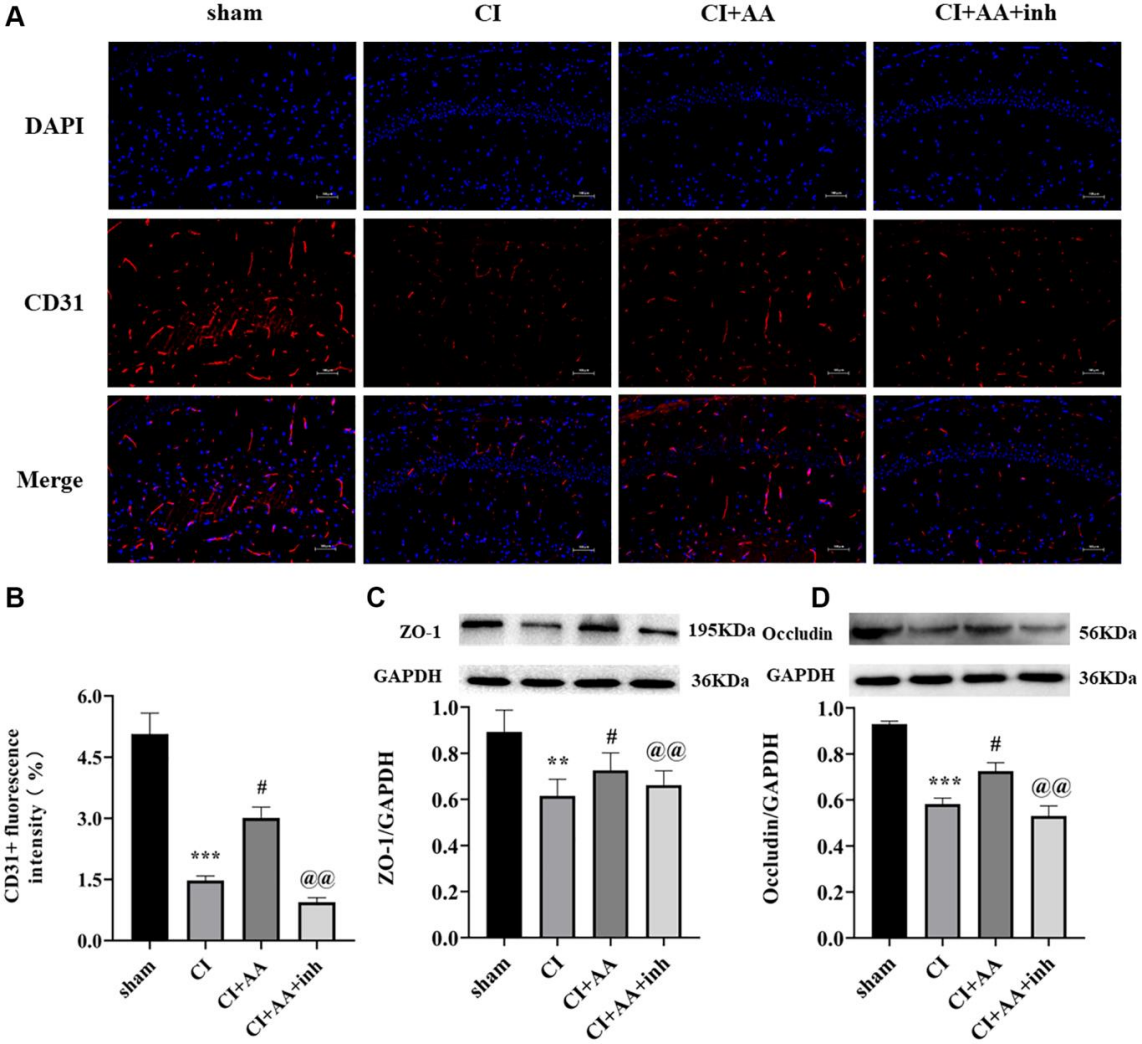
**Alisol A upregulated the expression of CD31, ZO-1 and Occludin after CI.** (**A**) Representative image of CD31 in the hippocampal CA1 region by using immunofluorescence staining. 200×, scale bar is 100 μm. (**B**) Quantitative analysis of CD31 fluorescence intensity. *n* = 3. (**C**) Western blot showing the expression of ZO-1 and quantitative analysis of the ratio of ZO-1 to GAPDH. (**D**) Western blot showing the expression of Occludin and quantitative analysis of the ratio of Occludin to GAPDH, *n* = 4. Data are shown as the mean ± SD. ^**^P < 0.01, ^***^P < 0.001 compared with the sham group, ^#^*P* < 0.05 compared with the CI group, ^@@^*P* < 0.01 compared with the CI+AA group.

CD31 is an angiogenesis marker that is mainly expressed in BMECs and is used to quantify the number of capillaries (Figure 5A). The expression of CD31 was decreased in the CI group (*P* < 0.001), alisol A treatment dramatically increased the expression of CD31 (*P* < 0.05), and the inhibitor abolished the effect of alisol A on CD31 expression (*P* < 0.01) (Figure 5B). ZO-1 and Occludin are important components of tight junction proteins (TJs), which are associated with BBB integrity and permeability. ZO-1 and Occludin were significantly decreased in the CI group (*P* < 0.01, *P* < 0.001), and alisol A treatment upregulated the expression of ZO-1 and Occludin (*P* < 0.05, *P* < 0.05), indicating that alisol A effectively protected the structure of the BBB by reducing the destruction of TJs. The AKT inhibitor reversed the effect of alisol A (*P* < 0.01, *P* < 0.01) (Figure 5C, 5D). The results confirmed that alisol A protected BMECs and TJs, which contributed to the protection of BBB integrity.

### Alisol A promoted neuronal survival in the hippocampus by downregulating the BAX/Bcl-2 ratio after CI

**Figure 6:**
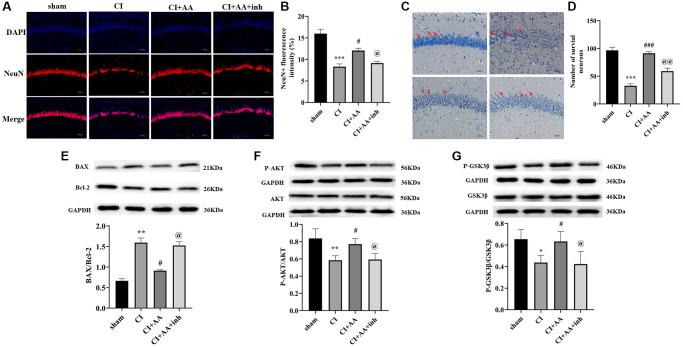
**Alisol A reduced neuronal apoptosis in the hippocampus by downregulating the BAX/Bcl-2 ratio and activating the AKT/GSK3β pathway after CI.** (**A**) Representative image of NeuN in the hippocampal CA1 region by using immunofluorescence staining. 200×, scale bar is 100 μm. (**B**) Quantitative analysis of NeuN fluorescence intensity, *n* = 3. (**C**) The neuronal morphology of the hippocampal CA1 region was observed by Nissl staining. 400×, scale bar is 50 μm. Red arrows show the morphology of neurons in different groups. (**D**) Quantitative analysis of intact neuronal cells in the hippocampal CA1 region, *n* = 3. (**E**) Western blot showing the expression of BAX and Bcl-2 and quantitative analysis of the BAX/Bcl-2 ratio. (**F**) Western blot showing the expression of p-AKT and AKT and quantitative analysis of the ratio of p-AKT to AKT. (**G**) Western blot showing the expression of p-GSK3β and GSK3β and quantitative analysis of the ratio of p-GSK3β to GSK3β, *n* = 4. Data are shown as the mean ± SD. ^*^P < 0.05, ^**^P < 0.01, ^***^P < 0.001 compared with the sham group, ^#^*P* < 0.05, ^###^*P* < 0.001 compared with the CI group, ^@^*P* < 0.05, ^@@^*P* < 0.01 compared with the CI+AA group.

The expression of NeuN (Figure 6A) in the hippocampal CA1 region of the CI group was markedly decreased compared with that in the sham group (*P* < 0.001), while alisol A treatment increased the expression of NeuN (*P* < 0.05), and the inhibitor decreased the expression of NeuN (*P* < 0.05) (Figure 6B). Nissl staining was applied to detect surviving neurons in the hippocampal CA1 region (Figure 6C). In the sham group, neuronal cells were arranged densely and neatly in the hippocampus. In contrast, the neuronal cells were arranged in a disorderly manner and even disappeared in the CI group (*P* < 0.001). Alisol A treatment restored the destruction of neurons during CI. The number of Nissl neurons in the alisol A group was higher than that in the CI group (*P* < 0.001) and inhibitor group (*P* < 0.01) (Figure 6D). Bcl-2 plays a role in promoting survival of neurons by inhibiting the expression of BAX. The BAX/Bcl-2 ratio in the CI group was higher than that in the sham group (*P* < 0.01), and the ratio in the alisol A group was lower than that in the CI group (*P* < 0.05) and AKT inhibitor group (*P* < 0.05) (Figure 6E). The results suggested that alisol A may promote neuronal survival in the CA1 region of the hippocampus by downregulating the BAX/Bcl-2 ratio.

### Alisol A activated the AKT/GSK3β pathway after CI

To verify whether the AKT/GSK3β pathway is involved in the protective effects of alisol A, we detected the expression of phosphorylated AKT (Ser473) and GSK3β (Ser9). Phosphorylation of AKT and GSK3β was markedly decreased in the CI group (*P* < 0.01, *P* < 0.05). Alisol A promoted the phosphorylation of AKT and GSK3β (*P* < 0.05, *P* < 0.05). The phosphorylation levels of AKT and GSK3β were reversed following treatment with the AKT inhibitor (*P* < 0.05, *P* < 0.05) (Figure 6F, 6G). This result indicated that alisol A may exert protective effects on the neurovascular component of the hippocampus by activating the AKT/GSK3β pathway.

## DISCUSSION

CI has seriously endangered the health of elderly individuals. The neurovascular integrity in the hippocampus is critical for learning and memory, which is susceptible to CI causes microglia-mediated inflammation and neurovascular disruption in the hippocampus, which is associated with cognitive impairment. Neurovascular impairment after CI primarily occurs in the hippocampal CA1 region and is often accompanied by cognitive impairment [20]. It is urgent to seek promising therapeutic strategies for neurovascular improvement with CI.

Alisma Orientale and its active constituents have been proven to have anti-inflammatory and vascular protective effects and have been clinically used to treat cardiovascular-related diseases [21]. Alisol A, as the active ingredient isolated from Alisma Orientale, its effect on cerebrovascular diseases is rarely reported. It has been reported that alisol A exerted anti-obesity effect by ameliorating hyperlipidemia and inflammation state [22]. Alisol A could be detected in rat cerebral tissue after oral administration [23], indicating that alisol A could exert some effects on improving cerebrovascular diseases. Moreover, alisol A has been reported to exert vascular protection, attenuate the expression of proinflammatory cytokines, and increase the expression of phospho-AKT [24]. AKT is one of the core therapeutic targets of the mechanism of action of alisol A. Phospho-AKT exerts protective effects in CI by inactivating GSK3β [25]. AKT/GSK3β pathway is closely related to the inhibition of inflammation and apoptosis and the protection of vascular endothelium. Activation of AKT/GSK3β promotes neuronal survival in the hippocampus, reduces glial inflammation, and maintains BBB integrity after CI, demonstrating that the AKT/GSK3β pathway plays an important role in neurovascular protection [26]. We speculated that alisol A-mediated neurovascular protection might be associated with the activation of AKT/GSK3β. The present study explored the neurovascular protection of alisol A, focusing on neurons, glial cells and BMECs. Our study is the first to show that alisol A has a protective effect on neurovasculature. We found that alisol A improved neurological deficits and cognitive impairment, inhibited glial activation, protected BMECs and TJs, promoted the survival of neurons and BMECs, and ameliorated neurovascular disruption in the hippocampus. Moreover, an AKT inhibitor reversed the protective effects of alisol A, which suggested that activation of the AKT/GSK3β pathway may participate in the neurovascular protection of alisol A.

MRS has been used to detect metabolic information in CI [27]. We first observed changes in neuronal loss and glial activation following ischemia according to the changes in NAA, Cho and MI levels, which may be associated with neurovascular dysfunction. Decreased NAA has been identified as a strong signal for neuronal loss [28]. The Cho concentration in glial cells is higher than that in neurons and may be used as a marker of gliosis [19]. MI is also a marker of glial proliferation [29]. Both Cho and MI levels increased after ischemia, indicating overactivation of glia after ischemia. Interestingly, AKT is a crucial regulator of cell energy metabolism, including cholinergic function, neurochemical differentiation, neuroprotection and inhibition of gliosis [30]. It was confirmed that activation of AKT regulated the levels of MI, Cho and NAA [18]. In this study, we found that CI induced the upregulation of Cho and MI and the downregulation of NAA. Interestingly, alisol A decreased Cho and MI concentrations in the hippocampal CA1 region while increasing the level of NAA, and an AKT inhibitor reversed this tendency. The results indicated that the regulatory effect of alisol A on hippocampal neuronal/glial metabolism might be related to the AKT/GKS3β pathway.

TEM results showed that the neurovascular ultrastructure was destroyed after CI, which manifested as neuronal morphological destruction, thickened capillary wall edema, and swelling of astrocytic end-foot processes in the hippocampus, which was consistent with a previous study [31]. Swollen astrocytic end-foot processes and perivascular edema are involved in the pathological process of BBB breakdown [32]. As expected, we found that treatment with alisol A improved the nuclear membrane of neurons, mitochondrial cristae, swelling of astrocyte end-feet and endothelial cells, while the neurovascular protective effect was eliminated by an AKT inhibitor.

We further investigated the underlying mechanism of neurovascular changes. Microglia and astrocytes are overactivated after CI, inducing inflammatory dysfunction [33]. Reactive astrogliosis produces large amounts of proinflammatory factors and damages neurovascular homeostasis [34]. Microglia-mediated inflammation affects basal lamina integrity and leads to increased permeability of the BBB, ultimately resulting in significant neurovascular disruption [35]. A previous study showed that the activation of AKT/GSK3β inhibited the proinflammatory “M1” phenotype of microglia and then downregulated IL-6, IL-1β and TNF-α levels [36–38]. The present study showed that alisol A significantly inhibited the expression of GFAP and Iba1 and reduced the overactivation of glial cells. In addition, the levels of the inflammatory factors IL-6 and IL-1β also decreased after alisol A intervention. However, the effects of alisol A could be attenuated by inhibition of AKT at the same time, further confirming that the AKT/GSK3β pathway was involved in the anti-inflammatory effect of alisol A.

The release of inflammatory cytokines induced by overactivated glial cells has been demonstrated to further damage the BBB [39]. CD31 (an angiogenesis marker), mostly expressed in BMECs, plays a pivotal role in protecting BBB integrity [40]. The activation of the AKT/GSK3β pathway exerted an angiogenic effect, and raised the CD31 positive microvessel number, while the inhibition of AKT inhibited angiogenesis, which was consistent with our study [41]. In addition, TJs form a virtually impermeable barrier between BMECs, which is relevant in maintaining BBB integrity. Studies have indicated that activation of AKT/GSK3β reduces the disruption of ZO-1, Occludin and Claudin-5 by downregulating the expression of inflammatory factors [42]. In our study, alisol A treatment upregulated the expression of CD31, ZO-1 and Occludin and promoted vascular and endothelial protection in the hippocampus, whereas the effects were attenuated by an AKT inhibitor, further revealing that alisol A may protect the BBB through regulation of the AKT/GSK3β pathway.

Overactivated glial cells also aggravate neuronal apoptosis in the hippocampal CA1 region after CI [43]. In our study, NeuN immunofluorescence and Nissl staining showed that alisol A treatment promoted neuronal survival in the hippocampus after CI. In addition, we found that alisol A decreased the ratio of BAX/Bcl-2, which may be what promotes neuron survival. The effect of alisol A was eliminated by inhibition of AKT. Therefore, we further explored the underlying mechanism. Bcl-2 promotes neuronal survival by inhibiting the expression of the proapoptotic factor BAX [44], while BAX is involved in neuronal apoptosis by triggering mitochondrial dysfunction [45]. AKT inhibits inflammation and apoptosis by inactivating GSK3β, and GSK3β promotes translocation of this proapoptotic protein to mitochondria in neurons undergoing apoptosis by directly phosphorylating BAX on Ser163 [46]. The BAX/Bcl-2 ratio is increased by downregulating the phosphorylation levels of AKT and GSK3β [47]. Furthermore, activating the AKT/GSK3β pathway has been proven to mediate the survival of hippocampal CA1 neurons after CI [48]. Therefore, the results suggest that alisol A may promote the protective effect of alisol A on neuronal survival in the hippocampus and decrease the BAX/Bcl-2 ratio by activating the AKT/GSK3β pathway.

The AKT/GSK3β pathway plays an important role in the protective effects of alisol A on neurons and the BBB. Notably, the inhibition of AKT decreased the phosphorylation level of GSK3β and almost reversed the neurovascular protective effects of alisol A, which was consistent with our hypothesis. Our study is the first to show that alisol A has a protective effect on cerebrovascular. The present study also showed that AKT/GSK3β pathway was involved in the neurovascular protective effect of alisol A, which is another novelty different from other studies.

In conclusion, this study showed that alisol A alleviated neurovascular injury, which was mainly attributed to the activation of the AKT/GSK3β pathway by raising the phosphorylation of AKT and GSK3β.

## CONCLUSIONS

Alisol A could exert an important role in neurovascular protection and alleviate cognitive impairment through activation of the AKT/GSK3β pathway in CI mice.
